# LncRNA AC136007.2 alleviates cerebral ischemic-reperfusion injury by suppressing autophagy

**DOI:** 10.18632/aging.203369

**Published:** 2021-08-13

**Authors:** Na Liu, Aini Peng, Haiyan Sun, Yuansu Zhuang, Ming Yu, Qun Wang, Jinping Wang

**Affiliations:** 1 Department of Neurology, The First People’s Hospital of Zhenjiang, Zhenjiang 212000, Jiangsu Province, China; 2 Department of Neurology, Jilin Provincial FAW General Hospital, Changchun 130000, Jilin Province, China; 3 Department of Neurology, Affiliated Hospital of Jiangsu University, Zhenjiang 212000, Jiangsu Province, China

**Keywords:** lncRNA, ischemic stroke, OGD/R, MCAO, autophagy

## Abstract

Differential expression and diagnostic significance of the long noncoding RNA (lncRNA) AC136007.2 has been reported in patients with acute ischemic stroke (AIS). However, its role on disease progression and outcome remains unclear. Here, we employed an oxygen-glucose deprivation/reperfusion (OGD/R) model in neuronal SH-SY5Y cells and performed middle cerebral artery occlusion (MCAO) surgery in rats to investigate the function of AC136007.2 in ischemia-reperfusion (I/R) injury. AC136007.2 expression was determined by RT-qPCR and cell viability was examined using CCK-8, Edu, LDH, and apoptosis assays. Pro-inflammatory cytokine expression was assessed using ELISA. OGD/R downregulated AC136007.2 expression in SH-SY5Y cells, decreased viability by inducing apoptosis, and stimulated secretion of TNF-α, IL-6, and IL-1β. In turn, lentivirus-mediated AC136007.2 overexpression significantly reversed these phenomena. LC3 immunofluorescence and western blotting analyses of LC3-I/II and Beclin-1 expression and AMPK/mTOR phosphorylation status showed that AC136007.2 suppressed autophagy in SH-SY5Y cells via inactivation of AMPK/mTOR signaling. Notably, incubation with the AMPK activator AICAR abolished the pro-survival effect of AC136007.2 upon OGD/R treatment. Importantly, intraventricular injection of AC136007.2 significantly reduced cerebral infarction and brain edema in MCAO rats, as shown by TTC staining and water content measurements. We conclude that AC136007.2 alleviates cerebral I/R injury by suppressing AMPK/mTOR-dependent autophagy.

## INTRODUCTION

Stroke is a major cause of long-term disability and ranks as the second cause of death in the world [1]. In the past few years, the burden of stroke in China has increased, with an estimated 2.4 million new cases and 1.1 million stroke-related deaths per year [2]. Ischemic stroke (IS), which is caused by cerebral vascular occlusion, accounts for nearly 80% of all strokes. Currently, intravenous thrombolysis and thrombectomy are the only effective treatments for IS [3, 4]. However, the clinical approach to cerebral ischemia-reperfusion injury (CI/RI) after restoration of blood supply remains unresolved. Therefore, the discovery of new therapeutic targets provides novel ideas for improving treatment efficiency.

Long noncoding RNAs (lncRNAs) conform a large class of RNA transcripts over 200 nucleotides in length. With the development of sequencing techniques and bioinformatics, numerous studies have revealed the regulatory role of lncRNAs in cellular physiological processes [5–7]. Several lncRNAs were found to be dysregulated in IS, both in patients and in animal models. Stroke models *in vitro* and *in vivo* suggested in turn important roles for some of these transcripts in regulating the extent of ischemia-reperfusion (I/R) injury [8–10]. Recently, differentially expressed lncRNAs were profiled in patients with acute ischemic stroke (AIS), and diagnostic efficiency was reported for one of the downregulated lncRNAs, AC136007.2, through receiver operating characteristic (ROC) curve analysis [11]. To evaluate the involvement of AC136007.2 in IS-related I/R injury, in this study we assessed its regulatory activity on cell viability, inflammatory marker expression, and infarct severity through oxygen-glucose deprivation/reperfusion (OGD/R) challenge in neurons and middle cerebral artery occlusion (MCAO) in an IS rat model.

## RESULTS

### AC136007.2 overexpression decreases OGD/R-induced death in SH-SY5Y cells

**Figure 1:**
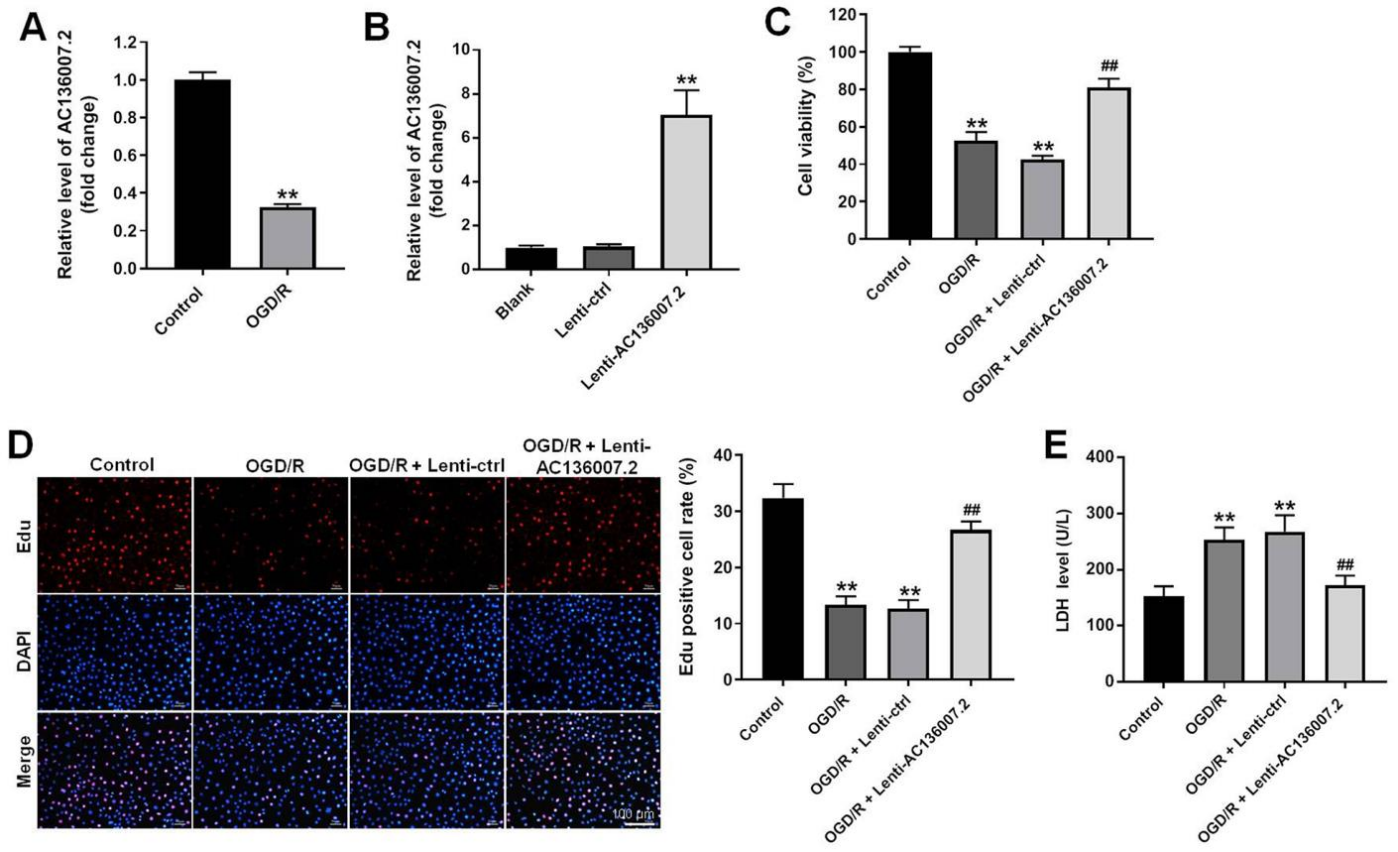
**Overexpression of AC136007.2 decreases OGD/R-induced cell death in SH-SY5Y cells.** (**A**) Relative AC136007.2 expression level was evaluated by RT-qPCR in SH-SY5Y cells incubated in normoxic (control) conditions and after OGD/R treatment. (**B**) RT-qPCR analysis of AC136007.2 expression after transfection with lenti-AC136007.2 or its negative control. (**C**) Cell viability was determined by the CCK-8 assay. (**D**) Cell proliferation was determined by EdU assay. The rate of Edu-positive cells (histogram on the right side) was calculated from three random fields of view, with total cell numbers determined by DAPI staining. (**E**) LDH-based cytotoxicity analysis. **p<0.01 versus control group; ^##^ p<0.01 versus OGD/R group; n = 3.

To simulate cellular injury after IS, SH-SY5Y cells were exposed to a 4h/18h OGD/R challenge. Consistent with lncRNA profiling data from AIS patients [11], OGD/R significantly decreased the expression of AC136007.2 in SH-SY5Y cells (Figure 1A). Then the cells were transduced with lenti-AC136007.2 or a control lentivirus and transduction efficiency confirmed by RT-qPCR (Figure 1B). Subsequently, cell viability and proliferation were examined using various assays. The CCK-8 assay showed that OGD/R decreased SH-SY5Y cell viability, while AC136007.2 overexpression significantly reversed this effect (Figure 1C). In turn, EdU assays revealed that OGD/R inhibited cell proliferation, an effect also notably reversed upon transduction with lenti-AC136007.2 (Figure 1D). Consistent with these results, OGD/R-mediated cytotoxicity, reflected by increased cellular LDH levels, was significantly blocked by AC136007.2 overexpression (Figure 1E). The above results indicate that AC136007.2 expression alleviates OGD/ R-induced death of SH-SY5Y cells in culture.

### AC136007.2 overexpression alleviates OGD/R-induced apoptosis and pro-inflammatory cytokine synthesis in SH-SY5Y cells

**Figure 2:**
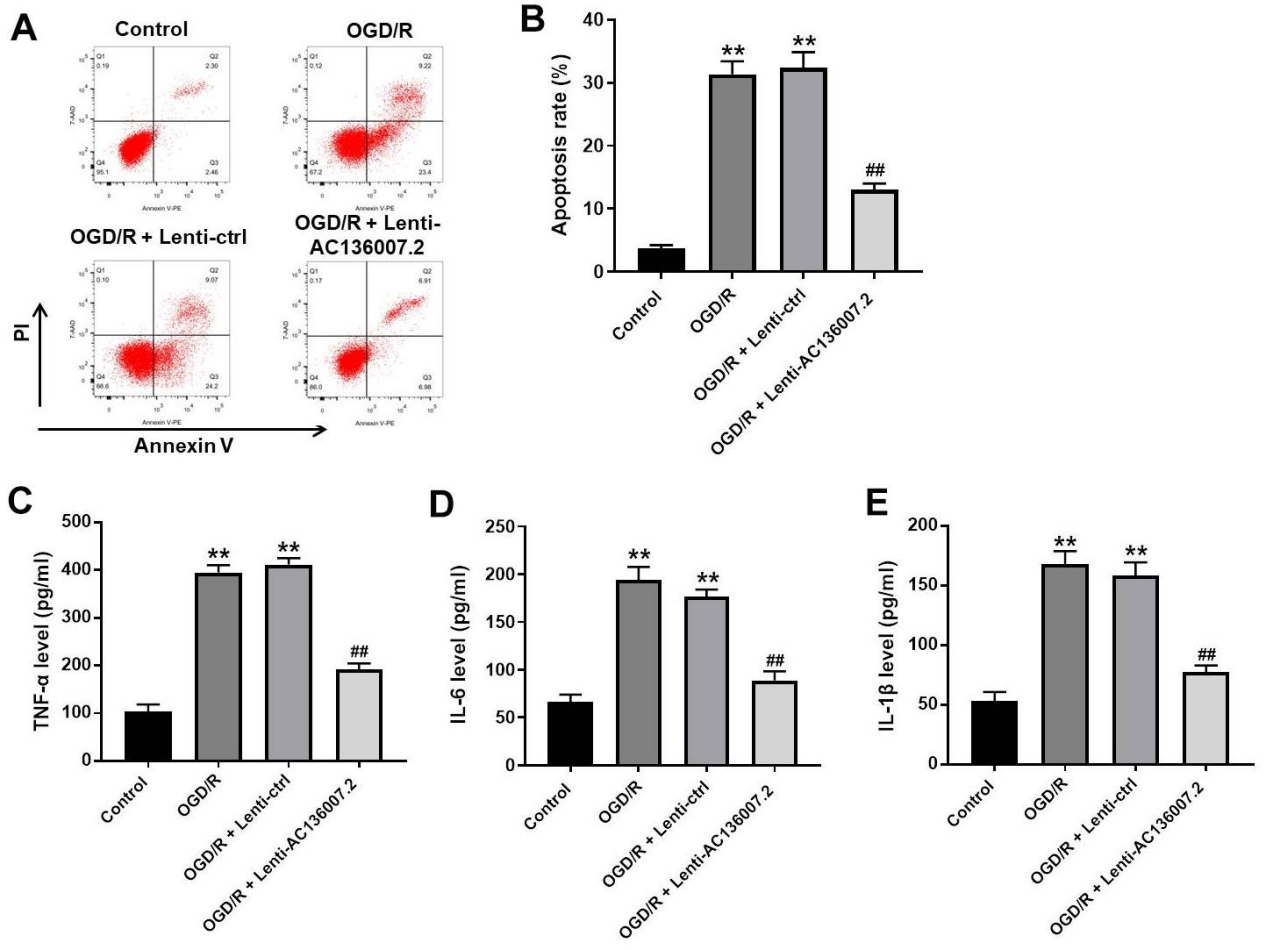
**Overexpression of AC136007.2 decreases OGD/R-induced apoptosis and inflammatory cytokine release in SH-SY5Y cells.** (**A**) Cell apoptosis was analyzed by flow cytometry after Annexin V/PI double staining. (**B**) Quantification of apoptosis rates from experiments like those shown in (**A**). (**C**–**E**) ELISA analysis of pro-inflammatory cytokine levels in cell culture supernatants. **p<0.01 versus control group; ^##^ p<0.01 versus OGD/R group; n = 3.

Apoptosis is an important cell death mechanism contributing to brain injury and neuronal death during and after IS [12]. In light of the pro-survival effects elicited by AC136007.2 in SH-SY5Y cells exposed to OGD/R, we employed flow cytometry to evaluate apoptosis in Annexin V/PI-stained cells. As expected, the apoptosis rate was dramatically increased by OGD/R. This phenomenon was, however, significantly mitigated after overexpression of AC136007.2 (Figure 2A, 2B). Neuroinflammation, resulting from excessive secretion of proinflammatory cytokines, contributes to neuronal injury and cellular dysfunction during IS [13]. Thus, we next used ELISA to examine proinflammatory cytokine production in SH-SY5Y cells. Results showed that OGD/R enhanced TNF-α, IL-6, and IL-1β levels in culture supernatants, whereas AC136007.2 overexpression significantly reduced this effect (Figure 2C–2E). The above data indicate that AC136007.2 expression alleviates apoptosis and decreases pro-inflammatory cytokine secretion induced by OGD/R in SH-SY5Y cells.

### AC136007.2 overexpression inhibits OGD/R-induced autophagy via AMPK-mTOR pathway inactivation in SH-SY5Y cells

**Figure 3:**
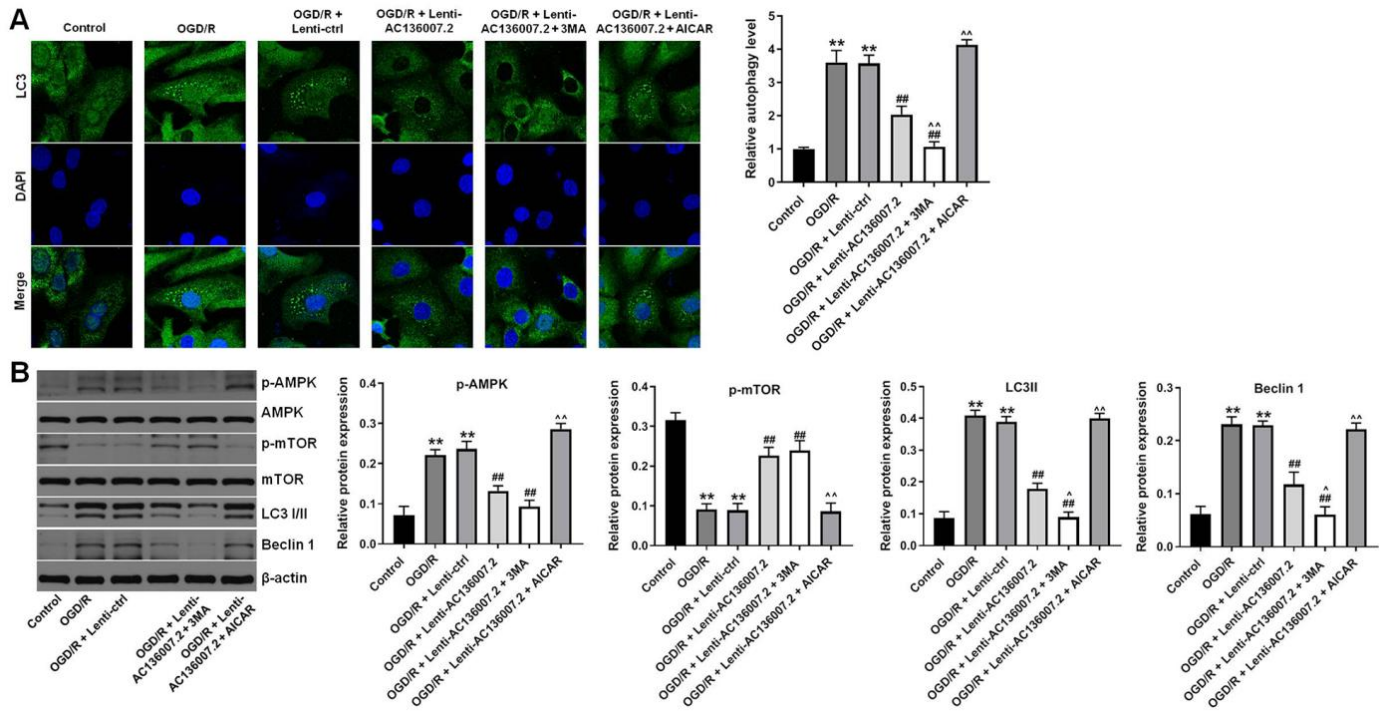
**Overexpression of AC136007.2 inhibits OGD/R-induced autophagy through inhibition of AMPK-mTOR signaling in SH-SY5Y cells.** (**A**) Representative images of LC3 immunofluorescence and semi-quantitative analysis of autophagy levels (right). Cell nuclei were counterstained with DAPI. (**B**) Western blotting analysis of LC3 I/II, Beclin 1, p-AMPK, and p-mTOR expression and corresponding densitometric quantification data. Signals were normalized to β-actin. **p<0.01 versus control group; ^##^ p<0.01 versus OGD/R group; ^^p<0.01 versus OGD/R + Lenti-AC136007.2 group; ^p<0.05 versus OGD/R + Lenti-AC136007.2 group; n = 3.

Increasing evidence suggests that autophagy is activated in various types of brain cells during IS [14]. Using immunofluorescence, we observed that OGD/R dramatically promoted the expression of LC3, a main marker of autophagosomes, in SH-SY5Y cells (Figure 3A). Notably, LC3 upregulation was largely inhibited by AC136007.2 overexpression, and this phenomenon was further enhanced after incubation of cells with the autophagy inhibitor 3-MA (Figure 3A). In contrast, treatment with the AMPK activator AICAR completely reversed the inhibitory effect of AC136007.2 on LC3 expression (Figure 3A). The suppressive action of AC136007.2 on OGD/R-induced autophagy was further confirmed by western blotting assays that showed, after OGD/R, decreased expression of LC3-I/II and Beclin 1 in cells overexpressing AC136007.2. We also examined the expression and phosphorylation status of key autophagy-associated kinases, namely AMPK and mTOR. Consistent with autophagy induction, OGD/R treatment increased the phosphorylation of AMPK and decreased the phosphorylation of mTOR. Overexpression of AC136007.2 significantly reversed the above phosphorylation changes in both enzymes, but failed to do so in AICAR-treated cells (Figure 3B). These findings suggest that AC136007.2 suppresses OGD/R-induced autophagy activation by regulating the activity of the AMPK-mTOR pathway.

### AC136007.2 alleviates OGD/R-induced injury through autophagy inhibition

**Figure 4:**
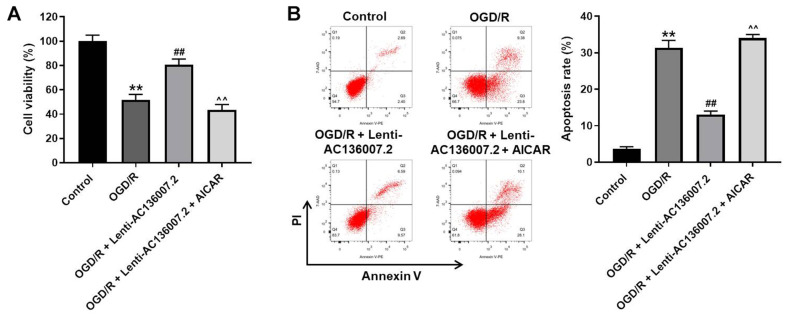
**Autophagy inhibition mediates the cytoprotective effect of AC136007.2 against OGD/R-induced injury.** (**A**) Cell viability was determined by the CCK-8 assay. (**B**) Cell apoptosis was analyzed by flow cytometry after Annexin V/PI double staining. **p<0.01 versus control group; ^##^ p<0.01 versus OGD/R group; ^^p<0.01 versus OGD/R + Lenti-AC136007.2 group; n=3.

Next, we explored whether the protective role of AC136007.2 in our neuronal OGD/R model is mediated by autophagy inhibition. Supporting this hypothesis, results of cell viability and apoptosis assays showed that pretreatment with AICAR abolished the protective effects of AC136007.2 on SH-SY5Y cells exposed to OGD/R (Figure 4A, 4B). These data indicate that AC136007.2 expression protects SH-SY5Y cells against OGD/R-induced death by inhibiting autophagy.

### AC136007.2 alleviates cerebral ischemic injury induced by MCAO in rats

**Figure 5:**
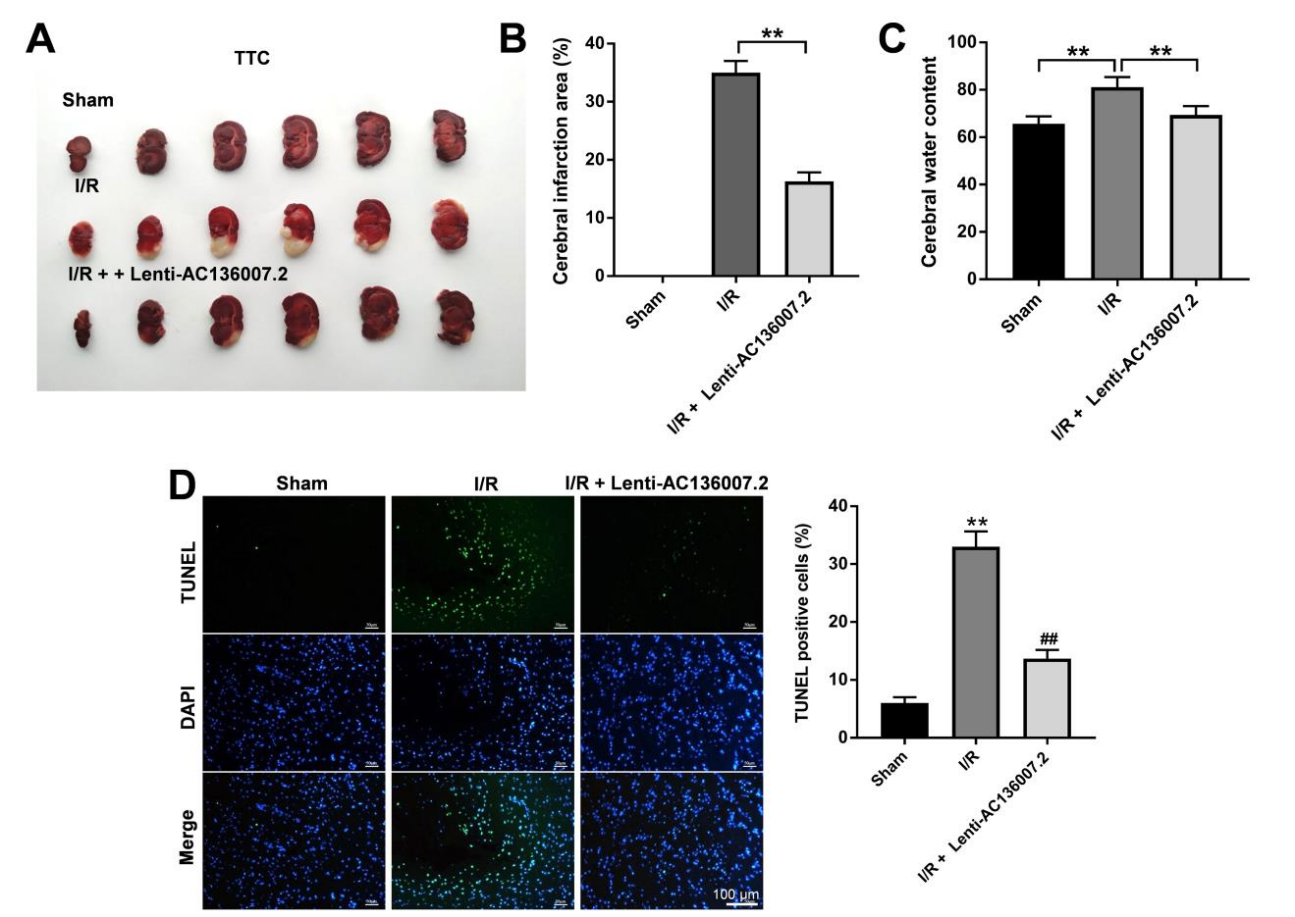
**AC136007.2 reduces cerebral infarction, brain edema, and brain cell death after MCAO in rats.** (**A**) Detection of brain infarction area by TTC staining. (**B**) TTC-based quantification of cerebral infarction (as percentage of the whole hemisphere). **p<0.01 versus I/R (MCAO) group; n=6. (**C**) Cerebral water content measurements. **p<0.01 versus I/R group; n=6. (**D**) TUNEL staining assessment of apoptosis in brain sections. **p<0.01 versus sham group; ^##^ p<0.01 versus I/R group; n=6.

To assess whether AC136007.2 expression confers protection against IS *in vivo*, a MCAO-I/R model was established in rats. A total of 54 rats, distributed into 3 groups (sham, I/R, and AC136007.2 + I/R; 18 rats/group), were included in this experiment. In the AC136007.2 + I/R group, lenti- AC136007.2 was injected into the brain right ventricle 24 h prior to MCAO surgery. MCAO lasted 2 h, and no deaths were recorded over the course of the study in any experimental group. The impact of ventricular AC136007.2 infusion on infarct size was evaluated by TTC staining 24 h after reperfusion. Results showed no obvious morphological alterations in the brains of sham-operated rats. Notably, the extent of cerebral infarction induced by MCAO was significantly reduced by pretreatment with AC136007.2 (Figure 5A, 5B). Consistent with this protective effect, the brain edema caused by MCAO was also significantly prevented after AC136007.2 administration (Figure 5C). In addition, we performed TUNEL staining to evaluate the extent of apoptosis in brain samples from the different experimental groups. Consistent with the above data, the number of TUNEL-positive cells was significantly reduced after AC136007.2 injection, compared to control MCAO rats (Figure 5D). These findings indicate that AC136007.2 expression plays a protective role against MCAO-induced cerebral infarction in rats.

### AC136007.2 inhibits MCAO-induced autophagy through inactivation of the AMPK-mTOR pathway

**Figure 6:**
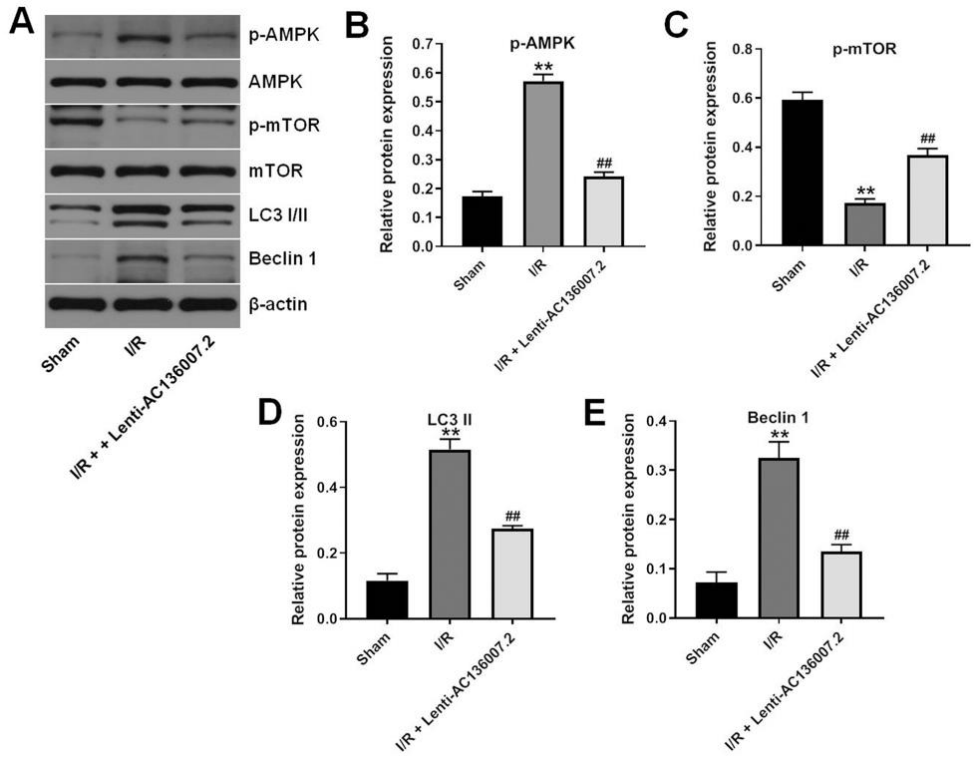
**AC136007.2 inhibits IS-induced autophagy by inactivating AMPK-mTOR signaling.** (**A**) Brain expression of LC3 I/II, Beclin 1, p-AMPK, and p-mTOR was evaluated by western blotting. (**B**–**E**) Quantification of western blotting results. Expression data were normalized to β-actin. **p<0.01 versus sham group; ^##^ p<0.01 versus I/R group; n=6.

Finally, we evaluated whether autophagy was activated in the brain tissue of MCAO rats. In agreement with the findings from our OGD/R model, western blotting results showed that MCAO induced upregulation of p-AMPK, LC-3 I/II, and Beclin 1, and downregulation of p-mTOR in the brain. Consistent with autophagy suppression, these changes were notably reversed by administration of AC136007.2 (Figure 6A–6E). These data suggest that AC136007.2 expression inhibits activation of the autophagy pathway in brain cells during AIS.

## DISCUSSION

Studies have shown that the expression profile of lncRNAs is significantly altered during the pathophysiological process of ischemic stroke [10, 11, 15]. Recently, Li et al. reported significant downregulation of circulating AC136007.2 levels in stroke patients and suggested that this lncRNA might be a potential marker of AIS [11]. Because the specific involvement of AC136007.2 in the pathogenesis of IS remains so far unclear, we modeled IS-associated I/R injury *in vitro* and *in vivo* to ascertain its role.

IS is a complex condition, associated with dysregulation of various pathophysiologic processes (e.g. cell proliferation, apoptosis, oxidative stress, and inflammation) that are influenced by numerous lncRNAs. Research has shown that lncRNAs may function as pro-apoptotic or anti-apoptotic agents to regulate cell survival during IS. For example, lncRNA-MEG3 was shown to be upregulated in human IS tissue samples, and its knockdown alleviated neuronal death during IS by reinstating miR-424-5p-mediated inhibition of Sema3A expression in MCAO mice [16]. In contrast, Wu et al. showed that lncRNA-N1LR inhibited apoptosis in OGD/R and MCAO models through inactivating p53 [17]. Our study suggests also a protective effect for lnc-AC136007.2, which promotes cell survival by inhibiting apoptosis in OGD/R-challenged neuronal cells.

Several lncRNAs were also shown to play an important regulatory role in I/R-induced neuroinflammation. For example, lncRNA-SNHG14 knockdown significantly attenuated the release of TNF-α induced by OGD in microglial BV-2 cells [18]. In turn, silencing of lncRNA-Malat1 was reported to potentiate the secretion of E-selectin, MCP-1, and IL-6 in brain microvascular endothelial cells, and to increase infarct size in MCAO mice [19]. An anti-inflammatory effect similar to that of Malat1 was thus detected in our study for AC136007.2, which decreased, upon overexpression, the secretion of pro-inflammatory cytokines induced by OGD/R.

An important finding in this study was that the neuroprotective action of AC136007.2 against I/R injury is critically mediated by inhibition of autophagy. Autophagy has emerged as a novel therapeutic target for stroke in recent years [14, 20]. A pioneer study by Puyal et al. in a focal ischemia model showed that intracerebroventricular injection of 3-MA after reperfusion reduced significantly (by 46%) brain infarction in rat pups [21]. This suggested that inhibition of autophagy provided neuroprotection during stroke. In recent years, the modulation of IS-induced autophagy has been shown to be influenced by various lncRNAs. For instance, overexpression of miR-378, a target of lncRNA-MEG3, inhibited neuronal autophagy and functional impairment in MCAO mice [22]. In turn, knockdown of lncRNA-MIAT reduced both apoptosis and autophagy in OGD/R-treated PC12 cells [23]. However, there are contradictory viewpoints regarding autophagy, as this phenomenon was proposed to attenuate cerebral I/R injury in some investigations [24, 25], Thus, this topic demands further research, both in *in vitro* and in animal models.

In conclusion, our results indicate that AC136007.2 expression promotes cell viability in both OGD/R-exposed neurons and in a rat MCAO model via autophagy inhibition. While these findings improve our understanding of the mechanisms by which lncRNAs regulate the pathophysiology of IS, further research is needed to identify the transcript(s) targeted by AC136007.2 and the specific regulatory interactions that influence autophagy activation/inhibition in the setting of IS.

## MATERIALS AND METHODS

### Cell culture and lentiviral transduction

The SH-SY5Y cell line was obtained from American Type Culture Collection (Manassas, VA, USA) and maintained in DMEM containing 10% FBS. Cells were incubated in a standard incubator at 37° C with 5% CO_2_. Lenti-AC136007.2 vector and its negative control were constructed by GenePharma (Shanghai, China), and transfected into 293T cells using Lipofectamine 2000 (Thermo Fisher Scientific, New York, NY, USA). SH-SY5Y cells were transduced with lentiviral particles secreted in the supernatant of 293T cells. After 24 h, the cells were used for downstream analyses.

### OGD/R model *in vitro*

To mimic OGD-induced injury, cultured SH-SY5Y cells were rinsed with PBS and the medium exchanged with glucose-free DMEM. The cells were then placed in a hypoxia incubator (95% N_2_ and 5% CO_2_) at 37° C [26]. After 4 h of OGD, the cells were transferred back to a conventional incubator and allowed to recover under normoxia in standard glucose-containing medium for 18 h.

### Real time-quantitative PCR (RT-qPCR)

A TRIpure kit (ELK Biotechnology, Wuhan, China) was used to extract total RNA from SH-SY5Y cells following the manufacturer’s protocol. After cDNA synthesis (EntiLink^TM^ cDNA Synthesis Kit; (ELK Biotechnology) PCR reactions were conducted on a StepOne™ PCR System (Applied Biosystems, Carlsbad, CA, USA) using ELK SYBR Green PCR SuperMix reagents. Primers for AC136007.2 were: sense: 5’-CCTCCCGTGCTACCCTTTAC-3’; antisense: 5’-CCTCTCCATTTCCAGGAACTG-3’. β-actin was used as the internal standard. Relative AC136007.2 expression levels were determined by the 2^−ΔΔCt^ method [27].

### Cell viability and cytotoxicity determinations

A CCK-8 kit (Keygen, Nanjing, Jiangsu, China) was used to assess cell viability. A lactate dehydrogenase (LDH) assay kit (Beyotime, Shanghai, China) was used to evaluate cytotoxicity. All assays were conducted according to the manufacturers’ instructions.

### 5-Ethynyl-20-deoxyuridine (EdU) assay

A BeyoClick™ EdU-594 kit from Beyotime was used to assess cell proliferation. SH-SY5Y cells were labeled with Edu reagent at 37° C for 2 h, and then fixed with 4% paraformaldehyde for 20 min. Nuclei were counterstained with DAPI. EdU-positive cells were counted in three random fields per well. Images were captured by a fluorescence microscopy (Leica, Wetzlar, Germany) and data was normalized to total cell numbers estimated by DAPI staining.

### Detection of pro-inflammatory cytokine expression

Levels of TNF-α, IL-6, IL-1β were determined with ELISA kits from ELK Biotechnology. In brief, the supernatants of SH-SY5Y cells were collected after the different treatments and analyzed following the instructions in the kits. Absorbance was measured at 450 nm with a plate reader.

### Cell apoptosis assay

SH-SY5Y cells were harvested and washed with cold PBS containing 0.5% FBS and 2 mM EDTA. After two additional washes in PBS, the cells were resuspended in binding buffer and stained with Annexin V-FITC/PI (Nanjing KeyGen Biotech Co Ltd., Nanjing, China) as per the manufacturer’s guidelines. The analysis was performed within 1 h on a flow cytometer (BD Biosciences, San Jose, CA) and apoptosis rates estimated with FlowJo 7.6 software.

### Immunofluorescence assay

SH-SY5Y cells were fixed with 4% paraformaldehyde for 20 min and permeabilized with 0.1% Triton X-100 for 20 min. BSA (4%) was then applied at room temperature for 30 min to block unspecific protein binding sites. Next, the cells were treated with an anti-LC3 antibody (1:400; Cell Signaling Technology; CST; Danvers, MA, USA) at 4° C overnight, followed by secondary antibody incubation for 1 h. Cell nuclei were counterstained with DAPI and images captured under fluorescence microscopy.

### MCAO model

Sprague-Dawley (SD) male rats (250–300 g) were purchased from Beijing Vital River (Beijing, China). The rats were housed in a standard humidity- and temperature-controlled room with a 12-h light/dark cycle. A total of 54 rats were randomly assigned into 3 groups (n=18 rats per group): sham, ischemia and reperfusion (MCAO; I/R), and AC136007.2 + I/R. The experimental protocols were approved by the Animal Ethics Committee of The First People’s Hospital of Zhenjiang and followed the recommendations of the NIH Guide for the Care and Use of Laboratory Animals.

MCAO surgery was performed on rats to establish an I/R model *in vivo*. To this end, the rats were anesthetized with 2.5% isoflurane and fixed onto a 37° C heating pad to maintain body temperature. After disinfection, a 2-cm midline incision was made in the neck to expose the left external carotid artery (ECA), which was then ligated where it branches into the into the lingual (LA) and the maxillary artery (MA). Then, a nylon monofilament coated with silicon was inserted through the ECA into the internal carotid artery (ICA) to occlude the right middle cerebral artery (MCA). Total distance was around 20–24 mm [28]. Blood supply of the brain was blocked for 2 h, followed by reperfusion for 24 h. Rats in the sham group received the same procedure without MCAO, while animals in the AC136007.2 + I/R group received a slow injection of lenti-AC136007.2 into the brain’s right ventricle 24 h prior to MCAO surgery. At the end of the reperfusion period all the rats were sacrificed to obtain brain samples.

### Measurement of cerebral infarction volume

Brain samples were cut into 2-mm-thick slices and incubated with 2% 2,3,5-triphenyltetrazolium chloride (TTC, Sigma-Aldrich, St. Louis, MO, USA) for 20 min at 37° C. After fixation in 4% paraformaldehyde for 24 h, images were taken by a digital camera, with red representing normal brain tissue and white representing infarct loci. The area of cerebral infarction was measured using NIH ImageJ software (Bethesda, MD, USA) and expressed as a percentage of the whole hemisphere.

### Measurement of brain water content

A wet/dry method was used to determine brain water content. Wet weight was first determined, immediately after excision. Next, the brain was dried at 110° C for 24 h and weighed again to determine dry weight. Brain water content was defined as the difference between wet and dry weight.

### TUNEL assay

TUNEL assay was performed using an *In Situ* Cell Death Detection Kit (Roche, Indianapolis, IN, USA) as per the manufacturer’s standard protocol. Nuclei were visualized with DAPI, and green fluorescence indicated TUNEL-positive cells. Images were captured from 3 random fields of view for each brain sample using fluorescence microscopy.

### Western blotting

Protein from SH-SY5Y cells or brain tissues was extracted using RIPA buffer containing protease and phosphatase inhibitors (Thermo, Wilmington, DE, USA). An equal amount of lysate for each sample was separated by 12% SDS-PAGE. Protein transfer onto PVDF membranes was performed with iBLOT2 (Invitrogen) for 6 min. Then the membranes were blocked with 5% BSA for 1 h, and incubated with primary antibodies at 4° C: p-AMPK (1:800), p-mTOR (1:800), AMPK (1:1000), mTOR (1:1000), LC3A/B (1:1000), LC3-B (1:1000), Beclin-1 (1:1000), and β-actin (1:5000) as loading control. After 1 h incubation with suitable HRP-conjugated secondary antibodies, an ECL kit (R&D, Minneapolis, MN, USA) was used to detect protein bands. All antibodies were purchased from CST (Danvers, MA, USA) and images were obtained using a Gel Imaging System (BioRad, Hercules, CA, USA).

### Statistical analysis

All experiments were repeated at least three times. The results were analyzed by GraphPad Prism software (La Jolla, CA, USA) and expressed as mean ± SD. A two-tailed Student’s *t*-test was applied to compare differences between two groups, and p < 0.05 indicated significance.

## References

[1] Virani SS, Alonso A, Benjamin EJ, Bittencourt MS, Callaway CW, Carson AP, Chamberlain AM, Chang AR, Cheng S, Delling FN, Djousse L, Elkind MS, Ferguson JF, et al, and American Heart Association Council on Epidemiology and Prevention Statistics Committee and Stroke Statistics Subcommittee. Heart Disease and Stroke Statistics-2020 Update: A Report From the American Heart Association. Circulation. 2020; 141:e139–596. 10.1161/CIR.0000000000000757 31992061

[2] Wang W, Jiang B, Sun H, Ru X, Sun D, Wang L, Wang L, Jiang Y, Li Y, Wang Y, Chen Z, Wu S, Zhang Y, et al, and NESS-China Investigators. Prevalence, Incidence, and Mortality of Stroke in China: Results from a Nationwide Population-Based Survey of 480 687 Adults. Circulation. 2017; 135:759–71. 10.1161/CIRCULATIONAHA.116.025250 28052979

[3] Meschia JF, Brott T. Ischaemic stroke. Eur J Neurol. 2018; 25:35–40. 10.1111/ene.13409 28800170

[4] Hankey GJ. Stroke. Lancet. 2017; 389:641–54. 10.1016/S0140-6736(16)30962-X 27637676

[5] Olivero CE, Martínez-Terroba E, Zimmer J, Liao C, Tesfaye E, Hooshdaran N, Schofield JA, Bendor J, Fang D, Simon MD, Zamudio JR, Dimitrova N. p53 Activates the Long Noncoding RNA Pvt1b to Inhibit Myc and Suppress Tumorigenesis. Mol Cell. 2020; 77:761–74.e8. 10.1016/j.molcel.2019.12.014 31973890 PMC7184554

[6] Meng Q, Wang K, Brunetti T, Xia Y, Jiao C, Dai R, Fitzgerald D, Thomas A, Jay L, Eckart H, Grennan K, Imamura-Kawasawa Y, Li M, et al. The DGCR5 long noncoding RNA may regulate expression of several schizophrenia-related genes. Sci Transl Med. 2018; 10:eaat6912. 10.1126/scitranslmed.aat6912 30545965 PMC6487854

[7] Li YP, Duan FF, Zhao YT, Gu KL, Liao LQ, Su HB, Hao J, Zhang K, Yang N, Wang Y. A TRIM71 binding long noncoding RNA Trincr1 represses FGF/ERK signaling in embryonic stem cells. Nat Commun. 2019; 10:1368. 10.1038/s41467-019-08911-w 30911006 PMC6433952

[8] Bao MH, Szeto V, Yang BB, Zhu SZ, Sun HS, Feng ZP. Long non-coding RNAs in ischemic stroke. Cell Death Dis. 2018; 9:281. 10.1038/s41419-018-0282-x 29449542 PMC5833768

[9] Dykstra-Aiello C, Jickling GC, Ander BP, Shroff N, Zhan X, Liu D, Hull H, Orantia M, Stamova BS, Sharp FR. Altered Expression of Long Noncoding RNAs in Blood After Ischemic Stroke and Proximity to Putative Stroke Risk Loci. Stroke. 2016; 47:2896–903. 10.1161/STROKEAHA.116.013869 27834745 PMC5127755

[10] Zhang J, Yuan L, Zhang X, Hamblin MH, Zhu T, Meng F, Li Y, Chen YE, Yin KJ. Altered long non-coding RNA transcriptomic profiles in brain microvascular endothelium after cerebral ischemia. Exp Neurol. 2016; 277:162–70. 10.1016/j.expneurol.2015.12.014 26746985 PMC4761283

[11] Li S, Zheng H, Chen L, Xu C, Qu X, Qin Z, Gao J, Li J, Liu J. Expression Profile and Potential Functions of Circulating Long Noncoding RNAs in Acute Ischemic Stroke in the Southern Chinese Han Population. Front Mol Neurosci. 2019; 12:290. 10.3389/fnmol.2019.00290 31849604 PMC6895137

[12] Naito MG, Xu D, Amin P, Lee J, Wang H, Li W, Kelliher M, Pasparakis M, Yuan J. Sequential activation of necroptosis and apoptosis cooperates to mediate vascular and neural pathology in stroke. Proc Natl Acad Sci USA. 2020; 117:4959–70. 10.1073/pnas.1916427117 32071228 PMC7060720

[13] Xu S, Lu J, Shao A, Zhang JH, Zhang J. Glial Cells: Role of the Immune Response in Ischemic Stroke. Front Immunol. 2020; 11:294. 10.3389/fimmu.2020.00294 32174916 PMC7055422

[14] Wang P, Shao BZ, Deng Z, Chen S, Yue Z, Miao CY. Autophagy in ischemic stroke. Prog Neurobiol. 2018; 163:98–117. 10.1016/j.pneurobio.2018.01.001 29331396

[15] He W, Wei D, Cai D, Chen S, Li S, Chen W. Altered Long Non-Coding RNA Transcriptomic Profiles in Ischemic Stroke. Hum Gene Ther. 2018; 29:719–32. 10.1089/hum.2017.064 29284304

[16] Xiang Y, Zhang Y, Xia Y, Zhao H, Liu A, Chen Y. LncRNA MEG3 targeting miR-424-5p via MAPK signaling pathway mediates neuronal apoptosis in ischemic stroke. Aging (Albany NY). 2020; 12:3156–74. 10.18632/aging.102790 32065781 PMC7066902

[17] Wu Z, Wu P, Zuo X, Yu N, Qin Y, Xu Q, He S, Cen B, Liao W, Ji A. LncRNA-N1LR Enhances Neuroprotection Against Ischemic Stroke Probably by Inhibiting p53 Phosphorylation. Mol Neurobiol. 2017; 54:7670–85. 10.1007/s12035-016-0246-z 27844279

[18] Qi X, Shao M, Sun H, Shen Y, Meng D, Huo W. Long non-coding RNA SNHG14 promotes microglia activation by regulating miR-145-5p/PLA2G4A in cerebral infarction. Neuroscience. 2017; 348:98–106. 10.1016/j.neuroscience.2017.02.002 28215748

[19] Zhang X, Tang X, Liu K, Hamblin MH, Yin KJ. Long Noncoding RNA Malat1 Regulates Cerebrovascular Pathologies in Ischemic Stroke. J Neurosci. 2017; 37:1797–806. 10.1523/JNEUROSCI.3389-16.2017 28093478 PMC5320610

[20] Puyal J, Clarke PG. Targeting autophagy to prevent neonatal stroke damage. Autophagy. 2009; 5:1060–61. 10.4161/auto.5.7.9728 19713756

[21] Puyal J, Vaslin A, Mottier V, Clarke PG. Postischemic treatment of neonatal cerebral ischemia should target autophagy. Ann Neurol. 2009; 66:378–89. 10.1002/ana.21714 19551849

[22] Luo HC, Yi TZ, Huang FG, Wei Y, Luo XP, Luo QS. Role of long noncoding RNA MEG3/miR-378/GRB2 axis in neuronal autophagy and neurological functional impairment in ischemic stroke. J Biol Chem. 2020; 295:14125–39. 10.1074/jbc.RA119.010946 32605923 PMC7549047

[23] Guo X, Wang Y, Zheng D, Cheng X, Sun Y. LncRNA-MIAT promotes neural cell autophagy and apoptosis in ischemic stroke by up-regulating REDD1. Brain Res. 2021; 1763:147436. 10.1016/j.brainres.2021.147436 33745924

[24] Yao X, Yao R, Huang F, Yi J. LncRNA SNHG12 as a potent autophagy inducer exerts neuroprotective effects against cerebral ischemia/reperfusion injury. Biochem Biophys Res Commun. 2019; 514:490–96. 10.1016/j.bbrc.2019.04.158 31056262

[25] Li Z, Li J, Tang N. Long noncoding RNA Malat1 is a potent autophagy inducer protecting brain microvascular endothelial cells against oxygen-glucose deprivation/reoxygenation-induced injury by sponging miR-26b and upregulating ULK2 expression. Neuroscience. 2017; 354:1–10. 10.1016/j.neuroscience.2017.04.017 28433650

[26] Fallarini S, Miglio G, Paoletti T, Minassi A, Amoruso A, Bardelli C, Brunelleschi S, Lombardi G. Clovamide and rosmarinic acid induce neuroprotective effects in *in vitro* models of neuronal death. Br J Pharmacol. 2009; 157:1072–84. 10.1111/j.1476-5381.2009.00213.x 19466982 PMC2737666

[27] Livak KJ, Schmittgen TD. Analysis of relative gene expression data using real-time quantitative PCR and the 2(-Delta Delta C(T)) Method. Methods. 2001; 25:402–08. 10.1006/meth.2001.1262 11846609

[28] Longa EZ, Weinstein PR, Carlson S, Cummins R. Reversible middle cerebral artery occlusion without craniectomy in rats. Stroke. 1989; 20:84–91. 10.1161/01.str.20.1.84 2643202

